# Effect of *Ginkgo biloba* Extract EGb761 on Hippocampal Neuronal Injury and Carbonyl Stress of D-Gal-Induced Aging Rats

**DOI:** 10.1155/2019/5165910

**Published:** 2019-11-23

**Authors:** Juan Li, Yi-Chang Zhang, Gang Chen

**Affiliations:** ^1^Key Laboratory of Ministry of Education on Traditional Chinese Medicine Resource and Compound Prescription, Hubei Province Key Laboratory of Traditional Chinese Medicine Resource and Chemistry, Hubei University of Chinese Medicine, Wuhan 430065, Hubei, China; ^2^Hubei College of Chinese Medicine, Jingzhou 434020, Hubei, China

## Abstract

**Background:**

*Ginkgo biloba* extract is widely studied for antiaging activities, but little is known about its antiaging mechanism of protein carbonylation. In order to verify carbonyl toxification (stress) hypothesis of aging, we have investigated the effects of EGb761 on hippocampal neuronal injury and carbonyl stress of aging rats.

**Methods:**

Seventy-two Wister male rats were randomly assigned into six groups (*n* = 12), normal control (NC), model control (MC), vitamin E (VE, 60 mg/kg) group, EGb761 low doses (GBEL, 8.75 mg/kg), EGb761 moderate doses (GBEM, 17.5 mg/kg), and EGb761 high doses (GBEH, 35 mg/kg). Except the NC, the other groups were subject to subcutaneous administration of 0.5% D-gal (10 ml/kg/day) for 6 weeks to induce aging model. The study detected cognitive impairment in rats by Morris water maze test and the contents of superoxidase dismutase (SOD), malondialdehyde (MDA), total antioxidant capacity (T-AOC) by the related kits. The level of 4-hydroxy-2-nonenal (4-HNE) protein adducts in rat brain was detected, and the ultrastructure of hippocampus was observed.

**Results:**

The EGb761 treatment groups significantly improved the spatial learning and memory of rats. Moreover, EGb761 treatment could reduce hippocampal neuronal damage based on histopathological and ultrastructural observation. Further studies have proved that these activities are remarkably related with the reducing level of MDA, protein carbonyl and lipofuscin, and 4-HNE protein expression, as well as the increasing of SOD and T-AOC content. Furthermore, EGb761 improves telomerase activity by detecting telomerase activity in the brain of aging rats.

**Conclusion:**

Our data indicate that EGb761 is an effective agent against D-gal-induced hippocampal neuronal loss owing to its antioxidative as well as carbonyl stress properties. Meanwhile, the carbonylation hypothesis is confirmed that the high level of 4-HNE may cause age-related neurodegenerative disorders.

## 1. Introduction

Aging, occurring in the later stage of life, refers to a process of functional deterioration that affects normal homeostasis [1]. An aging-induced brain disorder, such as learning and memory decline, occurs due to impairment of synaptic transmission and changes in the neurotransmitter level [2]. Substantial evidence indicates that oxidative stress, including lipid peroxidation, protein oxidation, and DNA damage, plays an important role in age-associated diseases [3]. However, the levels of lipid peroxidation products, reactive carbonyl compounds, such as MDA or 4-HNE, are increased in aging tissues [4], and this increase is positively correlated with age. Moreover, it has been reported that impaired protein function, manifested as an increase in protein carbonyl, plays a crucial role in aging [5]. Protein carbonyl level has been adopted widely to evaluate oxidative damage and carbonyl-associated protein dysfunction [6].

The *Ginkgo biloba* L., described as a “living fossil,” has hardly undergone any progressive evolutionary changes over 200 million years [7]. The life span of this tree may extend as long as 10 centuries even longer. Medicinal extract from *Ginkgo biloba* leaves is presently one of the most commonly used drugs for the treatment of age-related disorders, such as deterioration of mental functions, failing memory, cerebral ischemia, dementia due to neuronal degeneration, and vascular deficiency [8]. Standardized extracts of *Ginkgo biloba* (EGb761) contain mainly about flavone glycosides, terpene lactones, proanthocyanidins, and organic acids [3]. Flavone glycosides, mainly quercetin, kaempferol, and isorhamnetin, account for 22–27%, Terpene lactones mainly contain ginkgolides and bilobalide. They account for 5–7% of the extract. The experimental and clinical studies have revealed that it possesses hepatoprotective [9] and photoprotective effects [10], DNA repair mechanism [11], and antioxidant [12] and anti-inflammatory activities [13]. Its pharmacological effect is attributed to multiple components present in the extract, and no individual component has been assigned to exert the effect [14].

In recent years, many articles have reported the protective effect of *Ginkgo biloba* extract. EGb761 can improve the diastolic dysfunction of aging cardiomyocytes in primary rat cardiomyocytes [15]. EGb761 protected against mitochondrial dysfunction in platelets and hippocampi of old mice [16]. EGb761 recovered the tau phosphorylation at the hippocampus and prefrontal cortex of hyperhomocysteinemia (HHcy) rats [17]. The chronic EGb treatment improved the short-term memory of middle-aged rats and might increase the survival of cortical neurons [18]. In conclusion, *Ginkgo biloba* extract has protective and therapeutic properties for aging. But there are many speculations and views about the mechanism and pathway.

In 1995, Yin and Brunk had put forward a carbonyl toxification (stress) hypothesis of aging, and the DM-carbonyl modification of biomolecules was hypothesized to be a central process of organismic aging [19]. Protein carbonylation, one of the most harmful irreversible oxidative protein modifications, is considered as a major hallmark of oxidative stress-related disorders [20]. Meanwhile, protein aggregation is a common pathological hallmark due to amino acid mutation and changes in the primary structure of the proteins in many degenerative diseases of the central nervous system, such as Alzheimer's, Parkinson's, and Huntington's disease [21]. Thus, the present study investigated the effect of EGb761 on hippocampal neuronal injury and protein carbonylation and discusses the relationship between aging and protein carbonylation.

## 2. Methods

### 2.1. Animal Procedures

*Ginkgo biloba* extract was identified by a validated HPLC procedure [16]. The animal study was conducted in accordance with the guidelines and approval of the Institutional Animal Ethical Committee. SPF Wistar male rats (190–210 g) were obtained from DongChuang Laboratory Animal Service Department, Changsha, China (HNACSDC 20101224). *Ginkgo biloba* extract comes from SPH Xing Ling Sci. & Tech. Pharmaceutical Co., Ltd., (lot: 150302). The rats were acclimated for 1 week before dosing in Experimental Animal Center of Hubei University of Chinese Medicine. Then, seventy-two rats were selected and randomly divided into six groups with 12 in each group: normal control (NC), model control (MC), vitamin E (VE, 60 mg/kg, Sigma, lot: T4389) group, EGb761 low doses (GBEL, 8.75 mg/kg), EGb761 moderate doses (GBEM, 17.5 mg/kg), and EGb761 high doses (GBEH, 35 mg/kg). EGb761 (standardized extract containing 24% flavonoids and 6% terpene lactones and less than 5 ppm of ginkgolic acids). In NC and MC group, each rats received only normal saline. The samples were given by oral gavage once a day for 6 weeks. In addition to the NC, in the other groups, 0.5% D-gal (10 ml/kg/day or 2 ml/200 g/day, Sinopharm Chemical Reagent Co., Ltd., lot: F20100812) was administrated subcutaneously for 6 weeks. The light : dark cycle is 12 : 12 dark/light. Turn on the light at 7:00 and turn off the light at 19:00. The trials experiment starts at 14:00 every day.

The rats were anesthetized and the chest was opened; the cardiac was perfused with 0.9% saline and then fixed with 4% paraformaldehyde after the liver became white. Brain, liver, and kidney tissues were taken out, and hippocampal tissues were separated on ice (2–4°C), and all tissues were washed by PBS twice to avoid contamination with the peripheral blood.

#### 2.1.1. Cognitive Impairment

The Morris water maze test was performed according to the procedures as described previously [22]. The apparatus consisted of a tank (120 cm in diameter and 50 cm in height) filled with water at approximately 25°C. The tank was divided into four quadrants and an escape platform was submerged 1.5 cm below the water surface in the center of the southeast quadrant. Oriented navigation trials were performed 4 times per day for 5 consecutive days. For each trial, the rat was gently placed in the water facing the tank wall at one of the four starting quadrant points. The rats swam freely until it found the platform within 120 s [23]. If failed, it was placed on the platform for 10 s. The time required for the rat to reach the platform (escape latency) was recorded to assess spatial learning ability. On the sixth day, the probe trial was made by removing the platform and allowing each rat to swim freely for 120 s. Time spent in the target quadrant, and the numbers of target crossings over the previous location of the target platform were recorded with a computerized video system (Chinese Academy of Medical Sciences, DigBehv-MR, China).

### 2.2. Measurement of Protein Carbonyl, MDA, and SOD Levels

Liver, kidney, and brain tissues were collected from rats, and the levels of total protein carbonyl, MDA, and SOD were determined photometrically in accordance with the manufacturer's protocol by using commercially available enzymatic assay kits (Nanjing Jiancheng Bioengineering Institute, China).

### 2.3. Detection of T-AOC, TE, and Lipofuscin Activity in the Brain

The levels of T-AOC in brain were determined by spectrophotometry in accordance with the enzymatic assay kits (Nanjing Jiancheng Bioengineering Institute, Nanjing City, P. R. China). Approximately 30 mg tissue was washed in ice-cold PBS for twice, telomerase was extracted, and finally homogenized in about 150 ml PBS. TE activity was measured in triplicate using a TE ELISA kit according to the manufacturer's instructions (Nanjing Sen Beijia Biological Technology Co., Ltd., Nanjing, China). The lipofuscin level was determined by the Sohal method [24]. In a nutshell, the cerebral cortex was about 200 mg with 2 ml chloroform-methanol (2 : 1) extracted, homogenized, and filtered, and then the residue was washed with chloroform-methanol. The combined filtrate, including the extract filtrate and the washed liquid, was added with chloroform-methanol to 5 ml, and the fluorescence intensity was measured.

### 2.4. Histopathological Examination

Hippocampal tissues were dissected, fixed with 10% neutral-buffered formalin, embedded in paraffin, sectioned at 3 *μ*m thickness, and then stained with hematoxylin and eosin (HE staining). Histological specimens were captured by digital microscope (XDS-18, Olympus, Japan).

### 2.5. Electron Microscopy

Brains were rapidly taken from the anesthetized rats. Hippocampal tissues were dissected and fixed with 2.5% phosphate-buffered glutaraldehyde. After that, fixed specimens should be dehydrated, infiltrated, embedded, sectioned, stained using standard techniques, and then examined by an FEI Tecnai G2 20 TWIN transmission electron microscope (USA).

### 2.6. Immunohistochemistry

Tissues of hippocampus, liver, and kidney were fixed, paraffin embedded, and sectioned using standard procedures. Then, the sections were deparaffinized in xylene, rehydrated in ethanol, and pressure cooked for antigen retrieval. The primary antibody anti-4-HNE (1 : 200, Abcam) was incubated for 60 min in a moist chamber at 37°C. Slides were then rinsed in PBS and subsequently incubated with the secondary antibody against anti-mouse antibody and the visualization of HRP (1 : 300, KPL). Then, the slides were washed, and the sections were developed in the enzyme substrate diaminobenzidine solution (DAB, Gene Company). Images of immunohistochemically stained sections were captured by the Olympus digital microscope. The integrated optical density (IOD) value was measured using Image-Pro Plus 6.0 software (Media Cybernetics).

### 2.7. Western Blot Analysis

The levels of 4-HNE adducts protein in brain, liver, and kidney tissues were analyzed using western blotting analysis. The total protein was extracted in an extraction buffer with protease and phosphatase inhibitor cocktails added (Servicebio Company, China), and the protein concentrations were determined using the BCA Protein Assay Kit (Beyotime, China) according to the manufacturer's instructions. Proteins were separated using 10% SDS-polyacrylamide gels and transferred onto a PVDF membrane. The blots were blocked with 5% nonfat milk in TBS and then incubated overnight at 4°C with anti-4-HNE antibody (1 : 300). After washing, the membrane was incubated for 1 h with secondary antibody (1 : 3000, KPL). The blot was then stripped and reprobed with *β*-actin (Santa Cruz) as the loading control. The membrane was washed 3 times and then visualized using Pierce ECL Western Blotting Substrate (Thermo Scientific).

### 2.8. HPLC Analysis

The HPLC analysis was carried on an Agilent Eclipse XDB-C18 (4.6 mm × 250 mm, 5 *μ*m) column with mobile phase consisting of 0.1% formic acid-water solution (A), acetonitrile (B), gradient elution (0–40 min, 10% B ⟶ 36%B; 40–45 min, 36% B ⟶ 50%B; 45–50 min, 50% B ⟶ 100% B; 50–55 min, 100% B; 55–56 min, 100% B ⟶ 10% B; 56–65 min, 10% B) was used. The flow rate was 1 mL·min^−1^. The detection wavelength was set at 245 nm, and the column temperature was 30°C [25]. Compared with the standard extracts of *Ginkgo biloba* leaves, the fingerprint of HPLC was used to determine the reliability of the extracts.

### 2.9. Statistical Analysis

Results were expressed as means ± SD for at least three independent experiments and were analyzed using the SPSS 18.0 software. Group differences in the escape latency in Morris water maze were analyzed using two-way analysis of variance (ANOVA) with repeated measures. The other data were analyzed with one-way ANOVA followed by post hochoc Tukey test. The value of *P* less than 0.05 was considered significant.

## 3. Results

### 3.1. EGb761 Improved the Spatial Learning and Memory of Aging Rats

**Figure 1:**
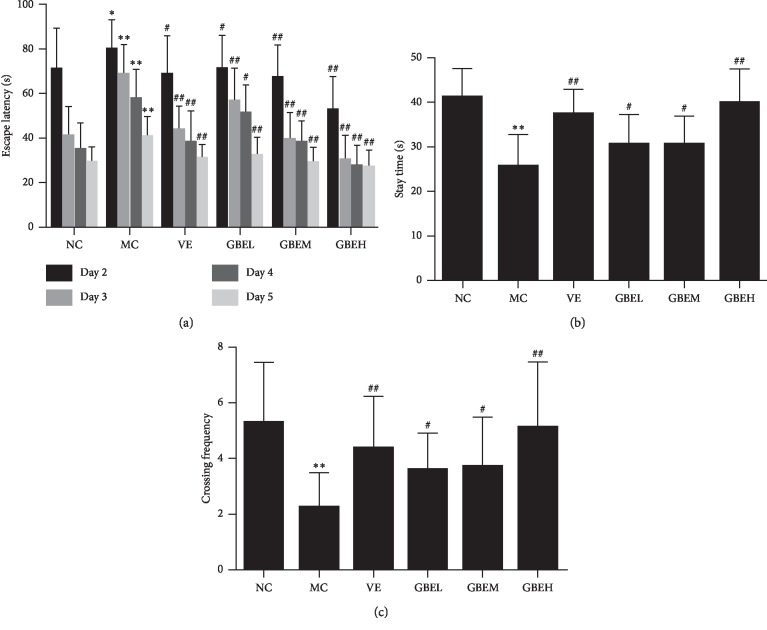
Effects of EGb761 on behavior capacity of aging rats. (a) The escape latency of the navigation experiment. (b) Target quadrant stay time. (c) The numbers of crossing over platform. All values were expressed as mean ± SD. Compared with the normal control group: ^*∗*^*P* < 0.05 and ^*∗∗*^*P* < 0.01; compared with the model control group: ^#^*P* < 0.05, ^##^*P* < 0.01, and *n* = 6.

In oriented navigation trials, all six groups were able to learn the task successfully, as evidenced by gradually reduced escape latency (Figure 1(a)). The escape latency in the MC group was markedly longer than that in the NC group (*P* < 0.01) (Figure 1(a)), suggesting that the learning and memory being impaired in the aging model rats. Meanwhile, the rats in EGb761 treatment groups showed notably shortened escape latency compared with MC group and had the similar level as the NC group (*P* < 0.01, Figure 1(a)).

In the probe trials, compared with the NC group, the rats in the MC group significantly shortened the time spent at the target quadrant (*P* < 0.01) (Figure 1(b)) and remarkably reduced the number of platforms crossing (*P* < 0.01) (Figure 1(c)). However, compared with the MC group, the rats in EGb761 groups prolonged the time spent at the target quadrant (*P* < 0.05) (Figure 1(b)) and significantly increased the number of platforms crossing (*P* < 0.05) (Figure 1(c)).

These results showed that aging model rats had impairments in spatial learning and memory, while the treatment of EGb761 could restore the behavior impairment caused by D-gal. Moreover, the aging rats treated with EGb761 showed relatively more effective on the behavioral capacity than treated with VE.

### 3.2. Effect of EGb761 on Protein Carbonyl, MDA, and SOD Levels in Aging Rats

**Figure 2:**
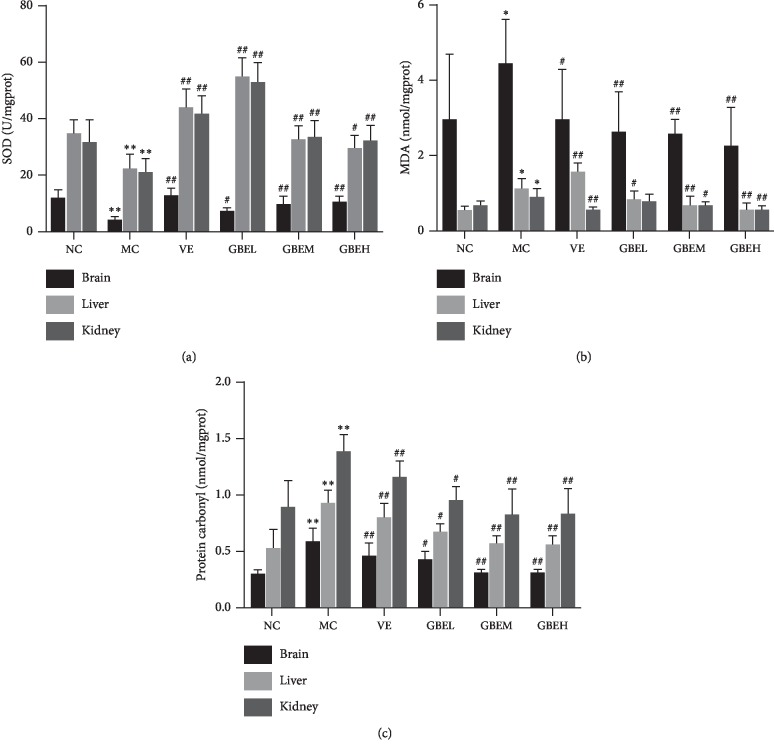
Effects of EGb761 on protein carbonyl, MDA, and SOD levels in aging rats. All values were expressed as mean ± SD. (a) SOD activity. (b) MDA activity. (c) Protein carbonyl level. Compared with the normal control group: ^*∗*^*P* < 0.05 and ^*∗∗*^*P* < 0.01; compared with the model control group: ^#^*P* < 0.05, ^##^*P* < 0.01, and *n* = 6.

As compared with the NC group, the rats in the MC group significantly decreased the SOD level but increased the MDA level in liver, kidney, and brain tissues (*P* < 0.05) (Figures 2(a) and 2(b)). Simultaneous treatment EGb761 with D-gal in rats caused a remarkable decrease in the activity of MDA level but increased the SOD level as compared with the MC group (*P* < 0.05) (Figures 2(a) and 2(b)). However, the results showed that administration EGb761H was similar to VE treatment.

As shown in Figure 2(c), the protein carbonyl level in the MC group was significantly increased compared with NC group (*P* < 0.01). Compared with MC group, the protein carbonyl level in the EGb761 group was significantly reduced (*P* < 0.05). Moreover, the results showed that administration EGb761H was similar to VE treatment.

### 3.3. Effect of EGb761 on the T-AOC, TE, and Lipofuscin in Aging Rats

**Figure 3:**
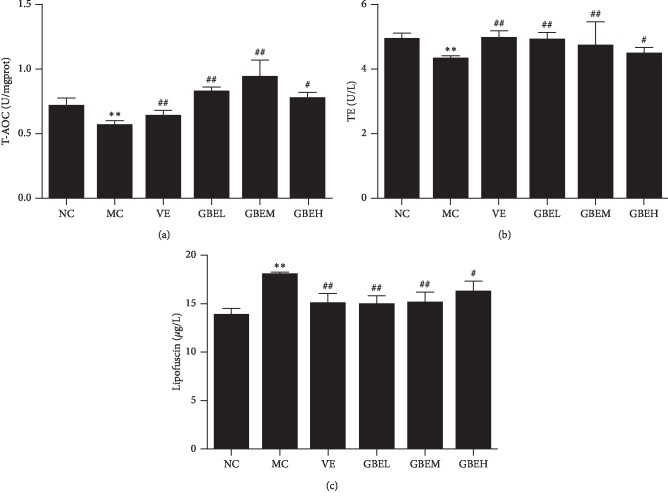
Effects of EGb761 on the T-AOC, TE, and lipofuscin in aging rats. All values were expressed as mean ± SD. (a) T-AOC on the brain. (b) TE on the brain. (c) Lipofuscin on the brain. Compared with the normal control group: ^*∗*^*P* < 0.05 and ^*∗∗*^*P* < 0.01; compared with the model control group: ^#^*P* < 0.05, ^##^*P* < 0.01, and *n* = 6.

In brain, there are significant differences in the levels of T-AOC, TE, and lipofuscin (*P* < 0.01) between the NC and MC group (Figure 3). However, T-AOC (Figure 3(a), *P* < 0.01) and TE (Figure 3(b), *P* < 0.01) levels were downgraded in the MC group, while the content of lipofuscin (Figure 3(c), *P* < 0.01) was increased in the MC group. Compared with the MC group, T-AOC and TE were upregulated, and lipofuscin was downregulated in the VE group and GBEH group (Figure 3, *P* < 0.05, *P* < 0.01).

### 3.4. Effect of EGb761 on Histopathological and Ultrastructural Changes

**Figure 4:**
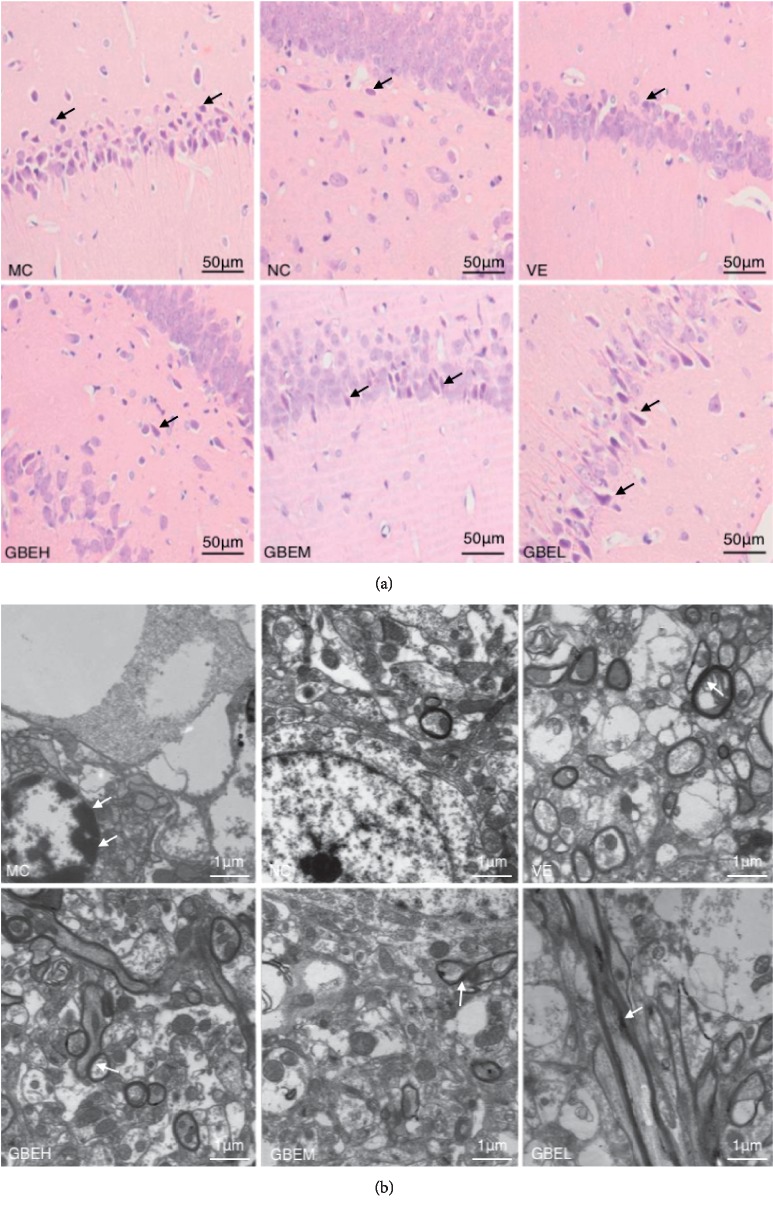
Effects of EGb761 on histopathological and ultrastructural changes in hippocampus of aging rats. (a) HE staining of CA1 region of hippocampus tissue (×400). (b) Transmission electron microscopy observation (×2500).

As shown in Figure 4(a), vertebral cell of control hippocampus showed normal morphology with round cell bodies and clear nuclei and nucleoli, most with vesicular nuclei of the CA1 region. After D-gal treatment, the vertebral cell layer of CA1 area was in disorder, and most cells were shrunken and distorted, with small dense nuclear remnants. However, degenerative changes and the shrunken cells were significantly attenuated by GBEH or VE administration. The GBEL or GBEM treatment did not significantly reduce hippocampal neuronal damage.

As shown in Figure 4(b), the NC neuron had a large round nucleus with the homogeneous karyoplasm, contained less lysosome, exhibited normal mitochondrial structure with clear cristae, and has well-developed rough endoplasmic reticulum, whereas after D-gal treatment, the MC neuron showed cytoplasmic vacuolization and organelles decreased with loosely myelin sheath. Mitochondria were enlarged and swollen and contained disrupted cristae and numerous lysosomes. Meanwhile, condensed or fragmented nuclei of glial cells, chromatin margination, and lipofuscin deposits were found in the MC group. Whereas, in the VE and GBEH group, the neuron showed obvious recovery on the ultrastructural damage, showing uniform electron density, well-developed mitochondrial structure, and plenty rough endoplasmic reticulum. The GBEL or GBEM treatment could more or less attenuate the hippocampal neuronal damage, showing better developed structure with less vacuolization and less lipofuscin granules than MC group.

### 3.5. Effect of EGb761 on Level of 4-HNE Protein Adducts

**Figure 5:**
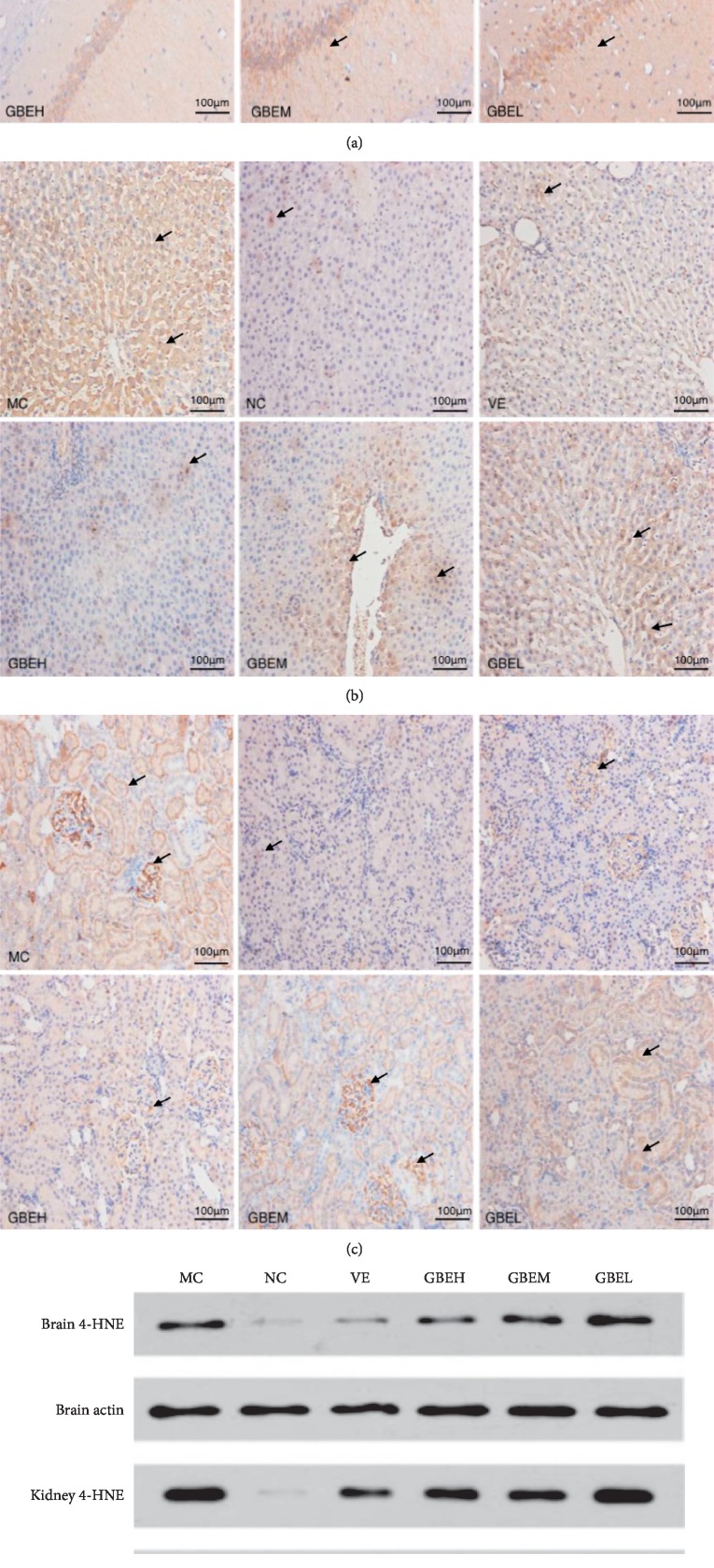
Effects of EGb761 on level of 4-HNE protein adducts in aging rats. (a) Hippocampus tissue. (b) Liver tissue. (c) Kidney tissue. (d) Western blot analysis, *n* = 3.

Immunohistochemistry and western blot results showed that the level of 4-HNE protein adducts were significantly increased in the MC group, compared with the corresponding NC group (Figure 5). However, simultaneous treatment VE or GBEH with D-gal in rats caused a decrease in the 4-HNE level, compared with the MC group. GBE1L or GBEM treatment could inhibit the 4-HNE level in some degrees, but with no significant difference. In addition, similar results could be seen in liver, kidney, and brain tissues.

### 3.6. HPLC of Extract of *Ginkgo Biloba* (EGb761)

**Figure 6:**
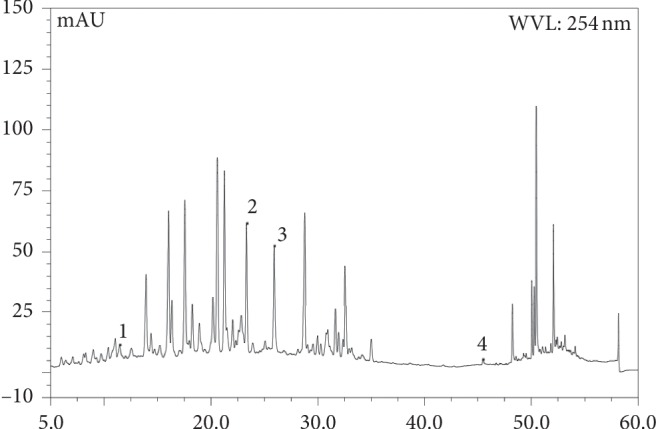
HPLC of EGb761: (1).rutin, (2) isoquercetin, (3) isorhamnetin-3-O-rutinoside, and (4) kaempferol.

The HPLC of EGb761 is similar to the fingerprint of EGb prepared by He et al. [25] at a wavelength of 254 nm. The results showed that EGb761 was the standard extract of *Ginkgo biloba* leaves (Figure 6).

## 4. Discussion

The study demonstrates significant effects of EGb761 administration in D-gal-induced oxidative stress associated with in rat liver, kidneys, and brain. The EGb761 administration effectively reduced MDA and protein carbonyls and increased SOD levels in three tissues. Moreover, it was effective to elevate the activity of reduced T-AOC and TE in brain and less lipofuscin deposition. Meanwhile, EGb761 administration significantly improved memory learning ability of aging rats in water maze. It can restore oxidative damage in hippocampal tissues and reduce the level of 4-HNE in three tissues.

In 1995, Yin and Brunk had put forward a carbonyl toxification (stress) hypothesis of aging, and the DM-carbonyl modification of biomolecules was hypothesized to be a central process of organismic aging [19]. With increase of protein carbonyls, the spontaneous carbonyl-amino crosslinking and accumulation were mostly irreparable changes associated with aging [26]. Thus, age-mediated brain disorders are closely associated with structural and functional changes in the hippocampus induced by protein carbonyls [21, 27]. These implicated that carbonyl stress impaired the function of proteins and then led to a series of age-related alterations.

The aging animal model induced by D-gal has been extensively used in the field of antiaging medicines. In animals, galactose is normally metabolized by D-galactokinase and galactose-1-phosphate uridyltransferase, but oversupply of D-gal may contribute to abnormal metabolism and cell damage, including a shortened life span, cognitive dysfunction, neurodegeneration, oxidative stress, decreased immune responses, production and accumulation of advanced glycation end products [28, 29], and an increase of protein carbonyl level [30]. The present study also found a significant increase of oxidative stress and carbonyl stress in liver, kidney, and brain tissues of D-gal-induced aging rats, evidenced by remarkable hippocampal neuronal loss, MDA and protein carbonyl production, and decline in SOD.

It is found that lipofuscin accumulates in many different tissues in a time-dependent manner [26]. Lipofuscin granules are mainly composed of oxidized proteins, lipid peroxidation products, and lesser amounts of carbohydrates and have the resistance to proteolytic degradation [31]. In the present study, the significant reduction of brain lipofuscin levels might be ascribed to decrease the content of MDA and protein carbonyl.

Biomarkers of cellular aging include telomeres and telomerase activity. To compensate for telomere shortening caused by cell division, telomerase is recruited to prolong to prevent cell apoptosis and senescence [32]. EGb761 can enhance telomerase activity in the current study, which is in agreement with the results of Dong et al. [33].

Long-term potentiation (LTP) has been extensively assumed as a cellular and molecular base for the deficit of spatial learning and memory in aged animals. Experiments showed that aging, D-gal-induced, showed impaired LTP in hippocampus [34]. The deficit in hippocampal LTP of aged rats was ameliorated by EGb761 administration [35].

4-HNE, specific diffusible end product of lipid peroxidation, is a highly reactive and has been shown to have toxic properties for numerous cells, especially neurons [36]. The production of 4-HNE has been implicated in the etiology of neurodegenerative diseases, cardiovascular diseases, cancers, diabetes, and acute lung injury [37]. This cytotoxicity is mainly due to its ability to form protein adducts via Michael addition with a vast number of targets [38]. In this study, we showed increased levels of 4-HNE in liver, kidney, and brain tissues of D-gal induced aging rats, compared with levels in the NC group. These implicated that 4-HNE impaired the function of proteins and then led to neuronal injury.

EGb761 has been used for years to treat Alzheimer's disease, dementia, vascular functions, and haemorrhage. In the recent study, we found that EGb761 could improve the spatial learning and memory and heal the hippocampal neuronal injury caused by D-gal through lowering the protein carbonyl and lipofuscin levels, reducing 4-HNE level, alleviating oxidative stress, increasing SOD, T-AOC levels, enhancing TE activity, and then prolonging aging process. It has been suggested to be an important way to maintain health and longevity. The results verified a viewpoint that carbonyl toxicition, high level of 4-HNE, may cause age-related neurodegenerative disorders.

## Data Availability

The data used to support the findings of this study are available from the corresponding author upon request.

## Additional Points

*Highlights. Ginkgo biloba* extract improves behavioral capability and heals hippocampal neuronal injury of aging rats. *Ginkgo biloba* extract alleviates oxidative stress and carbonyl stress. *Ginkgo biloba* extract inhibits 4-HNE expression.
